# Prompt and aggressive treatment of deep neck infection in neglected diabetic patient: A case report and literature review

**DOI:** 10.22088/cjim.14.2.406

**Published:** 2023

**Authors:** Stella Pravita, Sony Wibisono, Ivana Purnama Dewi

**Affiliations:** 1 Faculty of Medicine, Airlangga University, Surabaya, Indonesia; 2 Endocrinology Division, Department of Internal Medicine Dr. Soetomo General Hospital, Surabaya, Indonesia

**Keywords:** Diabetes mellitus, Deep neck infection, abscess

## Abstract

**Background:**

Deep neck infection (DNI) is an infection that occurs in the deep neck fascia and spaces commonly found in diabetic patients. Impaired immune system due to hyperglycemic condition in diabetic patients leads to different clinical presentations, prognosis, and management and therapy in this group of patients.

**Case Presentation:**

We reported a deep neck infection and abscess in a diabetic patient that resulted in acute kidney injury and airway obstruction. We have done CT-scan imaging that supported the diagnosis of a submandibular abscess. Prompt and aggressive treatment of DNI with antibiotics, blood glucose control, and surgical incision has exhibited a favorable outcome.

**Conclusion:**

Diabetes mellitus is the most common comorbidity among patients with DNI. Studies showed that hyperglycemia impaired bactericidal functions of neutrophils, cellular immunity, and complement activation. Aggressive treatment, including early incision and drainage of abscess, dental surgery to eradicate the source of infection, prompt empirical antibiotic administration, and intensive blood glucose regulation will result in favorable results without a prolonged hospital stay.

Deep neck infection (DNI) is an infection that occurs in the deep neck fascia and spaces. This infection can occur as an expansion of infections in different sites, namely in the pharynx, tonsils, sinuses, and most commonly, dental infection. As the disease progresses, DNI can cause deep neck abscess in which pus is accumulated in the interconnecting space and potential facial planes of the head and neck. The deep neck potential spaces are complex, connected structures (1, 2). Therefore, it remains a challenge to treat DNI. 

In recent years, the incidence of DNI has decreased over time due to the widespread use of antibiotics. However, this infection is still commonly found in people with comorbidities such as diabetes mellitus, alcoholism, or drug abuse (1). In diabetic patients, DNI is known to exhibit a unique clinical characteristic compared to the non-diabetic population. It may result in life-threatening conditions from airway obstructions, mediastinitis, emboli, and septic shock (3, 4). Although conservative antibiotic therapy is appropriate in some cases, most diabetic patients need a more comprehensive and aggressive treatment involving a multidisciplinary approach to avoid prolonged hospitalization, morbidity, and mortality. Here we reported a case of DNI in an uncontrolled diabetic patient. Aggressive treatment combined with infection eradication and well-regulated blood sugar resulted in a good outcome.

## Case Presentation

A 38-years-old woman came to primary health care with a complaint of a swollen right cheek for four days. The patient claimed to have no history of dental problems. Three days before her cheek was swollen, she admitted having her molar teeth manipulated with a toothpick due to food waste stuck in her right molar teeth. The patient showed swelling in her right cheek, high fever, pain in the swollen area, and difficulty opening her mouth or trismus. In the primary healthcare centre, the patient was diagnosed with abscess and diabetes mellitus (blood glucose was 350mg/dl at presentation) and treated with intravenous antibiotics and oral antidiabetic drugs for three days. However, the condition progressively worsened on the third day. The patient had intake difficulty and developed shortness of breath. Thus, she was immediately referred. 

**Figure 1:**
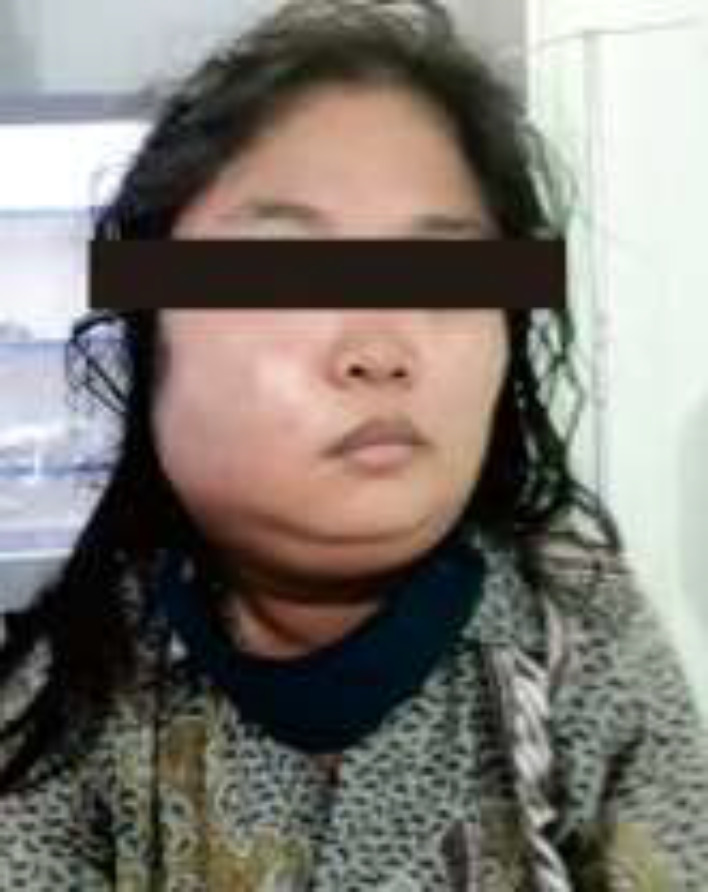
The patient's first clinical presentation, there is swelling and hyperemic in the right cheek to the neck area

**Figure 2:**
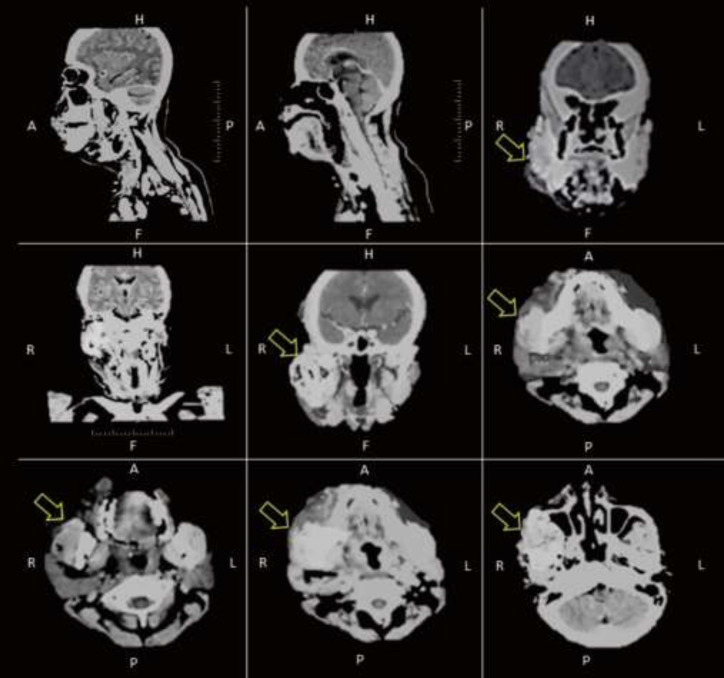
CT-scan imaging showed submandibular abscess with expansion to right parotid space, bilateral parapharyngeal space, epiglottis, retropharyngeal space, and upper-right neck area with prominent lymph nodes (yellow arrow)

The patient was fully awake in the emergency room with normal blood pressure and heart rate. She presented with an increased respiratory rate (22 times/minute), dyspnea, and fever (38.8^o^C). There was no abnormality in the heart and lung examination. The right buccal to the neck area was swollen and hyperemic (figure 1). There was a positive sign of pain and tenderness in the affected area. Abdomen and extremities were within normal limits. Complete blood count (CBC) results showed elevated leukocyte counts (20950/µ) with a higher proportion of neutrophils (88.2%) and a low proportion of lymphocytes (10.3%). Random blood glucose was 300 mg/dl with elevated creatinine level (1, 4 mg/dl) and normal blood urea nitrogen (15mg/dl). CT-scan imaging supported the diagnosis of submandibular abscess with expansion to right parotid space, bilateral parapharyngeal space, epiglottis, retropharyngeal space, and upper-right neck area with prominent lymph nodes (figure 2). The patient was then diagnosed with right submandibular-parapharyngeal-right parotid-retropharyngeal abscess due to infection in molar 46, 47 with comorbidities type 2 diabetes mellitus (T2DM) and pre-renal acute kidney injury (AKI). 

**Figure 3:**
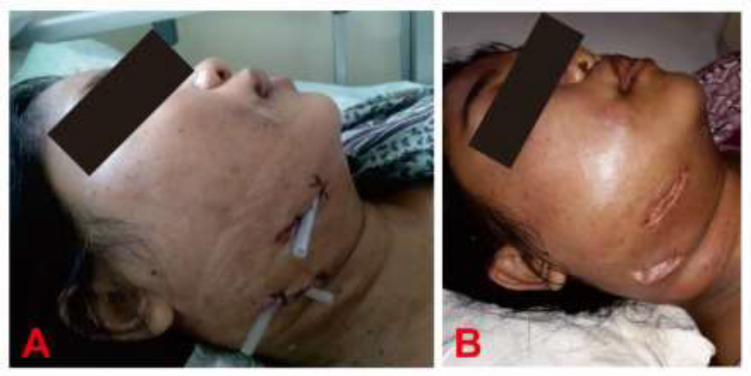
(A) Incision and drainage surgery for submandibular abscess (B) Abscess evaluation after 12 days of hospitalization with a better swelling presentation

Initial therapy included incision and drainage surgery for submandibular abscess and odontectomy for molars 46, 47 due to teeth decay (figure 3A). Intravenous fluid and per-enteral dietary intake were given post-operative. A broad-spectrum antibiotic, ceftriaxone (2x1g), was given with metronidazole (3x500mg) to eradicate the infection. Blood glucose was regulated through intravenous injection of insulin aspart 4 IU twice every hour until target blood glucose of 140-180mg/dl was achieved. Paracetamol injection was also administered (3x1gram) intravenously. The patient still experienced fever and trismus the next day; however, the creatinine and blood glucose decreased to 1mg/dl and 244 mg/dl, respectively. HbA1c result was 11.6% showing chronically uncontrolled blood glucose level. On the third day, the fever subsided (37.3^o^C), and bacterial culture returned positive for *Klebsiella pneumonia *sensitive to ceftriaxone. Therefore, antibiotic administration was continued. After eight days, the blood glucose returned to normal, although there was still active pus production from the parotid. The patient started per-oral dietary, and the insulin regimen was changed to the subcutaneous bolus of detemir 14 IU at night and Aspart 3x8 IU before the meal. On the 12^th^ day, there was no active pus production, blood glucose was normal, and the patient underwent abscess evaluation surgery and canal root removal for both molars (figure 3 B). After 15 days of care, the patient's blood glucose was well regulated, and there was no active pus production. Therefore, she was later discharged.

## Discussion

In adult patients, diabetes mellitus is the most common comorbidity found among patients with DNI. Due to defects in insulin secretion and bioactivation, chronic hyperglycemia affects the host's immune system. Studies showed that hyperglycemia impaired bactericidal functions of neutrophils, cellular immunity, and complement activation. Additionally, diabetic patients exhibit vascular insufficiency, promoting a higher risk of infection, including bacterial infection (4). The hyperglycemic state could also promote alteration in the oral area, including periodontal tissues, oral mucosa, salivary glands, and an oral neural function that can easily induce risk for caries (5). 

 In our case, the patient came to the primary health care with a clinical presentation resembling a parotid abscess, such as erythematous swelling in the right buccal area with fever, pain, and trismus as previously reported (6). Although the patient initially denied a history of dental problems, an odontogenic infection should be suspected since odontogenic infection is the major cause of DNI in diabetic and non-diabetic patients (2, 4, 7). Due to the hyperglycemic state, impaired immune and inflammatory responses are frequently found in diabetic patients. This condition was progressively turned into DNI despite the initial antibiotic and antidiabetic therapy in primary health care. One of the characteristics of DNI in diabetic patients is the involvement of more than one space in the abscess formation due to the inability of diabetic patients to localize an infection (4, 7, 8) with submandibular, parapharyngeal, tonsillar, and extended spaces as the most reported location (1, 2, 4).

 In an immunocompromised patient, infection in the oral cavity will easily go from maxilla or mandible lymphatic to the sublingual, submandibular, masticatory spaces and subsequently to the parapharyngeal space resulting in cervical lymphadenopathy that contributes to the airway obstruction (4). The exact course of the disease also happened in our case, where the buccal swelling expanded to the neck involving more than three spaces with characteristics suggesting DNI as previously reported (7). 

 Aside from the clinical presentation, the diagnostic work-up for abscess cases includes laboratory work-up, such as CBC, blood chemistry panel, and bacterial culture and sensitivity test (9). Leukocytosis with a prominent neutrophil count can be explicitly observed in diabetic patients with HbA1c≥7% (7, 8). Aside from leukocytes count, C-reactive protein can also be used as a parameter of multiple abscess formation as its value is significantly higher in multiple spaces abscess formation (1). From bacterial culture, *Klebsiella pneumonia* is the primary causative pathogen of DNI in diabetic patients, followed by *Streptococcus spp. *and *Staphylococcus spp., *which are also commonly found in non-diabetic patients (2-4, 8, 10). CT scan and ultrasonography are helpful imaging approaches to identify abscess formation from the early phase. CT with contrast enhancement is often the first modality chosen since it can easily differentiate soft tissue density within the gland (9). It is also helpful to differentiate cellulitis from an abscess (4). On the other hand, Sialography is preferable to diagnose chronic abscesses because its use is contraindicated for the acute phase due to the possibility of abscess rupture during the procedure (9). Ultrasonography is less invasive, low cost, easy to perform, and a widely used diagnostic modality (10). However, in some cases, this examination could not be performed in the acute phase. This examination put pressure on the lesion and induced pain in the patient, as we experienced in our case. It is widely known that diabetic patients have a higher risk of complications, including life-threatening complications and prolonged hospital stay due to DNI (1, 3, 4, 8). Therefore, treatment should be given more aggressively than in non-diabetic cases. In the presence of complications such as airway obstructions and dehydration, as seen in this patient, airway management and rehydration become the most important step in treating DNI. Tracheal intubation should be immediately performed, accounting for 12-16% of total DNI cases in severe airway obstruction cases (11). In DNI patients, oral intake difficulties could add significant morbidity as dehydration and renal injury occurs. Improvement in renal function was noted in our patient post-fluid therapy. Therefore, it should be noted that adequate fluid therapy, both intravenously or orally, is critical to the therapy algorithm (9, 12). 

In the case of abscess in multiple neck areas, surgical intervention remains the appropriate management. It should be done immediately, especially in patients with no response to previous antibiotic therapy, facial nerve involvement, and extensive abscess formation. Incision and drainage interventions are essential to remove pus and prevent further complications from airway obstruction, pneumonia, and sepsis. In addition to pus drainage, a multidisciplinary approach involving dental surgery specialists is also necessary to completely eradicate the source of infection (2, 4, 7-9). Alternatively, the medical approach, including empirical antibiotic administration, should also be started simultaneously. A broad-spectrum antibiotic covering gram-positive bacteria in combination with antibiotics against anaerobic bacteria should be initiated immediately as empirical therapy before the culture and sensitivity test are done. This approach was based on the polymicrobial bacteriology pattern of DNI, which includes aerobes, microaerophilic, and anaerobes. One of the most used drugs is the third-generation cephalosporin—ceftriaxone and metronidazole (2, 3, 7, 11). The antibiotic of choice can be changed after the culture and sensitivity test results.

Blood sugar regulation plays an important factor in the DNI therapy of diabetic patients. Studies showed that hyperglycemia conditions are related to neutrophil adherence, chemotaxis, phagocytic, and bactericidal impairment. However, hyperglycemia is often overlooked and results in severe and life-threatening infections. Therefore, tight and aggressive blood glucose control is necessary (3, 4). In our case, modest glucose therapy given by oral antidiabetic therapy in primary health care did not result in a good outcome.

 After an aggressive combination therapy of rehydration, early surgical intervention, insulin, and antibiotic administration at the hospital, the patient's condition gradually improved. There was no prolonged hospitalization for this patient. According to the previous reports, the average length of stay for DNI in diabetic patients is 10.5±6.3 days, 12.7 days, and 19.7±13.7 days (3, 4, 7). This patient was discharged after 16 days of care which falls into the average length of stay, without further complications.

We reported a case of DNI with diabetes mellitus and acute kidney injury in a female 38-year-old patient. The patient came with a chief complaint of buccal swelling that turned into a multi-space deep neck abscess with no response to the initial antibiotic and antidiabetic treatment. Diagnostic work-up, including laboratory evaluation, bacterial culture, sensitivity test, and CT scan, are necessary to deliver the appropriate treatment. Aggressive treatment, including early incision and drainage of abscess, dental surgery to eradicate the source of infection, prompt, empirical antibiotic administration, and intensive blood glucose regulation will result in favorable results without a prolonged hospital stay.
